# Molecular characterization of epithelial-mesenchymal transition and medical treatment related-genes in non-functioning pituitary neuroendocrine tumors

**DOI:** 10.3389/fendo.2023.1129213

**Published:** 2023-03-22

**Authors:** Joan Gil, Montserrat Marques-Pamies, Elena Valassi, Guillermo Serra, Isabel Salinas, Gemma Xifra, Paula Casano-Sancho, Cristina Carrato, Betina Biagetti, Gemma Sesmilo, Jennifer Marcos-Ruiz, Helena Rodriguez-Lloveras, Anna Rueda-Pujol, Anna Aulinas, Alberto Blanco, Cristina Hostalot, Andreu Simó-Servat, Fernando Muñoz, Marta Rico, Javier Ibáñez-Domínguez, Esteban Cordero, Susan M. Webb, Mireia Jordà, Manel Puig-Domingo

**Affiliations:** ^1^ Endocrine Research Unit, Germans Trias i Pujol Research Institute (IGTP), Badalona, Spain; ^2^ Department of Endocrinology, Research Center for Pituitary Diseases, Hospital Sant Pau, IIB-SPau, Department of Medicine, Universitat Autònoma de Barcelona, Barcelona, Spain; ^3^ Centro de Investigación en Red de Enfermedades Raras, CIBERER, Unit 747, Instituto de Salud Carlos III, Madrid, Spain; ^4^ Department of Endocrinology, Hospital Municipal de Badalona, Badalona, Catalonia, Spain; ^5^ Department of Endocrinology, Son Espases University Hospital, Palma de Mallorca, Spain; ^6^ Department of Endocrinology and Nutrition, Germans Trias i Pujol University Hospital, Badalona, Spain; ^7^ Department of Endocrinology, Josep Trueta University Hospital, Girona, Spain; ^8^ Centro de Investigación en Red de Diabetes y Enfermedades Metabólicas Asociadas (CIBERDEM), Pediatric Endocrinology Unit, Institut de Recerca SJS 39-57, Hospital Sant Joan de Déu, University of Barcelona, Esplugues, Spain; ^9^ Department of Pathology, Germans Trias i Pujol University Hospital, Badalona, Spain; ^10^ Department of Endocrinology, University Hospital Vall d’Hebron, Barcelona, Spain; ^11^ Department of Endocrinology, Dexeus University Hospital, Barcelona, Spain; ^12^ Department of Neurosurgery, Germans Trias i Pujol University Hospital, Badalona, Spain; ^13^ Department of Endocrinology, Hospital Universitari Mútua de Terrassa, Terrassa, Spain; ^14^ Department of Neurosurgery, Hospital de la Santa Creu i Sant Pau, Barcelona, Spain; ^15^ Department of Neurosurgery, Son Espases University Hospital, Palma de Mallorca, Spain; ^16^ Department of Neurosurgery, University Hospital Vall d’Hebron, Barcelona, Spain

**Keywords:** Epithelial-mesenchymal transition, non-functioning pituitary adenomas, somatostatin receptor ligands, dopamine agonists, somatostatin analogs

## Abstract

**Introduction:**

Different medical therapies have been developed for pituitary adenomas. However, Non-Functioning Pituitary Neuroendocrine Tumors (NF-PitNET) have shown little response to them. Furthermore, epithelial-mesenchymal transition (EMT) has been linked to resistance to medical treatment in a significant number of tumors, including pituitary adenomas.

**Methods:**

We aimed to evaluate the expression of EMT-related markers in 72 NF-PitNET and 16 non-tumoral pituitaries. To further explore the potential usefulness of medical treatment for NF-PitNET we assessed the expression of somatostatin receptors and dopamine-associated genes.

**Results:**

We found that *SNAI1, SNAI2*, Vimentin, *KLK10, PEBP1*, Ki-67 and *SSTR2* were associated with invasive NF-PitNET. Furthermore, we found that the EMT phenomenon was more common in NF-PitNET than in GH-secreting pituitary tumors. Interestingly, *PEBP1* was overexpressed in recurrent NF-PitNET, and could predict growth recurrence with 100% sensitivity but only 43% specificity. In parallel with previously reported studies, *SSTR3* is highly expressed in our NF-PitNET cohort. However, *SSTR3* expression is highly heterogeneous among the different histological variants of NF-PitNET with very low levels in silent corticotroph adenomas.

**Conclusion:**

NF-PitNET showed an enhanced EMT phenomenon. *SSTR3* targeting could be a good therapeutic candidate in NF-PitNET except for silent corticotroph adenomas, which express very low levels of this receptor. In addition, *PEBP1* could be an informative biomarker of tumor regrowth, useful for predictive medicine in NF-PitNET.

## 1 Introduction

Non-Functioning Pituitary Neuroendocrine Tumors (NF-PitNET) are defined as pituitary tumors that do not secrete biologically active hormones depicting specific hormonal symptoms. These neoplasms are the most common pituitary tumors in humans and when they come to medical attention, it is usually due to a mass effect and/or hypopituitarism (1–3). Histologically, NF-PitNET comprise a heterogeneous group of tumors arising from different pituitary cell lineages (4). The new WHO Classification of Pituitary Tumors 2022 divides NF-PitNET according to the cell of origin; however, it does not contain specific molecular information linked to treatment response prediction (5–7).

Nowadays, transsphenoidal surgery is the first-line treatment for most of these patients, whereas medical therapy -mostly temozolomide in aggressive tumors- and irradiation are reserved for patients that are not good surgical candidates or that have undergone unsuccessful surgery (5, 8). The recurrence rate of NF-PitNET in patients who underwent complete surgical resection is 20-25% at 10 years, while it rises up to 50-60% in case of partial removal (9, 10).

Therefore, there is a need to identify molecular targets in NF-PitNETs that clarify their biological behavior leading to the development of a more specific medical treatment. The current available targeted medical treatments for pituitary tumors are first and second generation somatostatin receptor ligands (SRLs), dopamine receptor D2 agonists (DA) and the alkylating agent temozolomide, usually reserved for very aggressive tumors (11, 12). Tumor shrinkage during therapy with either DA or SRLs has been previously reported in a variable percentage of NF-PitNET cases; however, response of NF-PitNET to medical treatment is still considered poor, insufficiently understood and nowadays unpredictable. Tumor reduction has only been observed in 12% of patients after octreotide treatment and in 28% of patients treated with DA therapy (13). Some authors suggested that SRLs treatment in NF-PitNET could only be useful for the stabilization of tumor growth without any significant effect on tumor shrinkage (14). Other authors have provided evidence favoring DA use when there is post-surgical residual tumor (15), independently of any other characteristic of the case.

Epithelial–mesenchymal transition (EMT) is a process that restructures the cell from an epithelial to a mesenchymal phenotype driven by a network of transcription factors (16). This process produces changes in post-translational regulation mechanisms and gene expression, leading to the loss of cell polarity and epithelial characteristics and the acquisition of increased migratory and invasive properties. EMT is not a binary process, and distinct intermediate cellular states have been reported (17). Epithelial-Mesenchymal Transition (EMT) has been linked to both the clinical course of NF-PitNET (18, 19) and resistance to medical therapy in pituitary tumors (20–23). Despite not being fully understood, several mechanisms have been described to explain the association of EMT and the lack of response to therapy in PitNETs. It has been proposed that EMT could disrupt alternative splicing in GH-secreting tumors (21) and that this is associated with a lack of response to somatostatin receptor ligands (23, 24). Another possible explanation is the involvement of the cytoskeleton, in particular, the actin binding protein filamin A that regulates localization, expression and signaling of SSTR2 and DRD2 in some PitNETs (25–29). Moreover, β-Arrestin 2 mediates the downstream effects of DRD2 in NF-PitNETs (30). The aim of our study is to elucidate the molecular landscape of NF-PitNETs for EMT-associated genes and genes related to the response to medical therapy with SRLs and DAs.

## 2 Materials and methods

### 2.1 Patients

This retrospective study enrolled 72 adult patients with NF-PitNET from 26 tertiary centers from all over Spain who underwent pituitary surgery and had tissue availability from 2014 to 2020. NF-PitNET was diagnosed by magnetic resonance image (MRI) of the sella turcica with a tumor visualized, in the absence of symptoms suggesting hormone hypersecretion and a biochemical confirmation of normal or hyposecretion of pituitary hormones. All tumors underwent surgical treatment. Furthermore, the pathologist selected a surplus of tumor to be embedded in RNAlater (Invitrogen, Carlsbad, CA, USA) for research purposes. All the tumors were naïve to medical treatment and radiotherapy. NF-PitNET invasiveness and size were established according to the preoperative MRI following the conceptual classification by Raverot (31). Tumor recurrence was considered when a new tumor image was detected in patients with no apparent postsurgical remnants or when a regrowth of a known remnant tumor was of sufficient clinical entity that required a second surgical intervention. The minimum surveillance time for considering a recurrence was 2 years, with a median of 5.32 ± 2.05 years for a total number of 58 patients. The clinical and neuropathogical characteristics of the cohort are described in **Table 1**. Sixteen non-tumoral pituitary samples from autopsies (8 samples) and organ donors (8 samples) were also analyzed as a non-pathological condition reference (mean age: 62.4 years ± 10.9; 37.5% females).

**Table 1:** Description of the cohort.

Patients Characteristics	
**Cohort (N)**	72
**Male/Female**	37/35
**Age (years)**	60.2 ± 13.5
Tumor Characteristics	
**Max. Diameter (mm)**	25.6 ± 8.9
**Extrasellar Extension**	59 (81.9%)
**Sinus Invasion**	34 (50.0%)
Presurgical Comorbidities	
**Hypopituitarism**	33 (47.8%)
**Headache**	31 (43.1%)
**Visual Alterations**	29 (40.3%)
Surgery Outcome	
**Cured**	30 (41.6%)
**Residual**	36 (50%)
**Exitus**	1 (1.4%)
**Missing**	5 (6.9%)
Postsurgical Comorbidities	
**Hypopituitarism**	36 (67.9%)
**Headache**	6 (11.3%)
**Visual Alterations**	12 (22.6%)
Tumor Subtypes	
**Silent ACTH +**	8 (11.1%)
**Silent FSH +/LH +**	21 (29.2%)
**Silent PRL +**	4 (5.6%)
**Negative tumors**	12 (16.7%)
**Plurihormonal tumors**	19 (26.4%)
**Missing**	8 (11.1%)

The molecular data of the GH-secreting adenomas used was obtained from our previous paper where the cohort is fully described (23). Briefly, a total of 57 (32 women) acromegaly patients from the REMAH cohort who underwent pituitary surgery and had tissue availability was used. Mean age was 45.74 ± 12.35, 82% of the tumors were macroadenomas, 68% presented extrasellar extension, with a mean tumor diameter of 19.49 ± 10.03.

The study was conducted in accordance with the ethical principles of the Declaration of Helsinki and implemented and reported in accordance with the International Conference on Harmonized Tripartite Guideline for Good Clinical Practice. The study was approved by the Germans Trias i Pujol Hospital Ethical Committee for Clinical Research (Ref.: EO-11-080 http://www.ceicgermanstrias.cat/index.html). The protocol and informed consent forms were approved by the institutional review board of the participating centers, independent ethics committee, and/or research ethics board of each study site. All patients provided written informed consent to participate in the study.

### 2.2 Immunohistochemical analysis

Formalin-fixed paraffin-embedded tumor samples were used for anterior pituitary hormone expression assessment. Growth hormone (GH), prolactin (PRL), thyroid-stimulating hormone (TSH), adrenocorticotropic hormone (ACTH), luteinizing hormone (LH) and follicle-stimulating hormone (FSH) expression were all tested with the corresponding antibodies, following local protocols for diagnostic assessment. Hematoxylin-eosin and reticulin staining, as well as cytokeratin, Ki-67, α-subunit and p53 immunolabeling, were also carried out in most of the cases as part of the standard pathological evaluation. Plurihormonal tumors were defined as tumors that showed immunoreactivity for more than one hormone that cannot be explained by normal cytophysiology or developmental mechanisms. We considered negative tumors those that did not express positivity for any of the hormones.

### 2.3 RNA isolation and reverse transcription

Representative fragments of the fresh tumor were selected by a pathologist and embedded in RNAlater (Invitrogen, Carlsbad, CA, USA) for 24 h, after which the samples were frozen at −80°C. Total RNA was isolated from pituitary adenomas using AllPrep DNA/RNA/miRNA Universal Kit (Qiagen, Hilden, Germany). We removed contaminating genomic DNA from RNA by treating samples with RNAse-free DNAse twice: the first time during the extraction of RNA following the manufacturer’s protocol and the second time, before the retrotranscription, for which ezDNase Enzyme (Invitrogen, Carlsbad, CA, USA) was used. The quantity and purity of DNA and RNA were measured using a NanoDrop™ 1000 spectrophotometer (RRID : SCR_016517, Thermo Fisher Scientific, Waltham, MA, USA). Integrity of the RNA was checked by agarose gel electrophoresis.

Five hundred nanograms of total RNA were reverse transcribed using SuperScript IV reverse transcriptase (Invitrogen, Carlsbad, California, USA), and random hexamers in a final volume of 20 uL according to the manufacturer’s protocol.

### 2.4 Quantitative polymerase chain reaction

Gene expression was quantified using Taqman assays (Applied Biosystems, Fosters City, California, USA). The genes analyzed were Somatostatin Receptor Subtype 2 (SSTR2, Hs00990356_m1), Somatostatin Receptor Subtype 3 (SSTR3, Hs00265633_s1), Somatostatin Receptor subtype 5 (SSTR5, Hs00990408_s1), short Dopamine Receptor D2 (DRD2) Isoform (Hs01014210_m1), long DRD2 Isoform (Hs01024460_m1), Arrestin Beta 1 (ARRB1, Hs00930516_m1), Pleiomorphic Adenoma Gene-Like 1 (PLAGL1, Hs00414677_m1), Raf Kinase Inhibitory Protein (RKIP/PEBP1, Hs01110783_g1), E-cadherin (CDH1, Hs01023894_m1), Ki-67 (MKI67, Hs01032443_m1), Ghrelin and Obestatin Prepropeptide (GHRL, Hs01074053_m1), Aryl Hydrocarbon Receptor Interacting Protein (AIP, Hs00610222_m1), Snail Family Transcriptional Repressor 1 (SNAI1, Hs00195591_m1), Snail Family Transcriptional Repressor 2 (SNAI2, Hs00950344_m1), Epithelial Splicing Regulatory Protein 1 (ESRP1, Hs00214472_m1), RAR Related Orphan Receptor C (RORC, Hs01076112_m1), N-Cadherin (CDH2, Hs00983056_m1), Twist Family bHLH Transcription Factor 1 (TWIST1, Hs00361186_m1) and Vimentin (VIM, Hs00958111_m1); furthermore, a custom assay was ordered for Intron 1 Ghrelin In1-GHRL (AJ89KWC). We tested six reference genes to normalize gene expression: Hypoxanthine Phosphoribosyltransferase 1 (HPRT1, Hs99999909_m1), Proteasome 26S Subunit ATPase 4 (PSMC4, Hs00197826_m1), Glucuronidase Beta (GUSB, Hs00939627_m1), TATA-Box Binding Protein (TBP, Hs00427621_m1), Mitochondrial Ribosomal Protein L19 (MRPL19, Hs01040217_m1) and Phosphoglycerate Kinase 1 (PGK1, Hs00943178_g1), and selected the last three reference genes based on their stability in our samples according to Chainy software (available on: http://maplab.imppc.org/chainy/) (32).

Quantitative polymerase chain reactions (qPCR) were carried out in a 7900HT Fast Real-Time PCR System (RRID : SCR_018060; Applied Biosystems, Fosters City, California, USA). We used TaqMan Gene Expression Master Mix (Applied Biosystems, Fosters City, California, USA), and the amplification reactions were performed in triplicate for each sample in a final volume of 10 uL in 384-well plates. To minimize the inter-assay variation, all the genes, including the reference genes, for each sample were analyzed in the same plate. To quantify relative gene expression we calculated a normalization factor for each sample based on the geometric mean of the selected reference genes, according to geNorm (RRID : SCR_006763, https://genorm.cmgg.be/) algorithms (33).

### 2.5 Statistical analysis

Descriptive results were expressed as mean ± standard deviation. Unsupervised hierarchical clustering was used to investigate the potential identification of patient’s response subgroups, based on their molecular expression profile. Differences between groups were compared using analysis of variance (Student’s t-test, Wilcoxon signed-rank test and Kruskal-Wallis analysis of variance as appropriate). Samples from all groups within an experiment were processed at the same time. The P values were two-sided, and statistical significance was considered when P < 0.05. All statistical analyses were performed using R version 4.2.1 (R Project for Statistical Computing, RRID : SCR_001905). Unsupervised hierarchical clustering was performed using the R package pheatmap (Pretty Heatmaps, https://CRAN.R-project.org/package=pheatmap). The graphical representation was done using package ggplot 2 (RRID : SCR_014601, Whickham https://CRAN.R-project.org/package=ggplot2) and the P values were added using the ggpubr package (‘ggplot2’ Based Publication Ready Plots, https://CRAN.R-project.org/package=ggpubr).

## 3 Results

### 3.1 Clinical description of the NF-PitNET cohort

We analyzed 72 patients with NF-PitNETs, 37 men and 35 women. The mean age was 60.3 years ± 13.5 and the mean follow-up was 915.7 ± 696.4 days. During follow-up, 6 patients with tumor remnants presented a recurrence after a mean of 759.8 days and 3 patients without visible remnants after surgery presented a recurrence after a mean of 783 days. Based on MRI, maximal tumor diameter did not show significant differences in tumors with or without extrasellar extension (p=0.22). However, maximal tumor diameter showed differences among tumors with sinus invasion (mean: 28.3 mm) and tumors without (mean: 23.3 mm) (p=0.02). The proportion of patients presenting headache and visual alterations fell after surgery (43.1% to 11.3%, and 40.3% to 22.6%, respectively in intrasellar and extrasellar tumors) while the proportion of patients presenting hypopituitarism increased after surgery (from 47.8% to 67.9%) (**Table 1**).

### 3.2 Association of EMT markers with clinical variables in NF-PitNETs

We analyzed the gene expression of different EMT-related markers and their relationship with clinical features of NF-PitNETs (**Table 2**). We found that SNAI1 and vimentin expression was associated with invasive tumors (p=0.049 and p=0.039, respectively) while E-cadherin expression was associated with non-invasive intrasellar tumors (p=0.047). Moreover, vimentin and SNAI1 overexpression showed an association with tumors presenting, respectively, headache and hypopituitarism before surgery (p=0.050 and p=0.048).

**Table 2:** Association between the relative expression of each EMT marker and different tumor characteristics.

Gene	Relative expression mean ± SE		P-value
TUMOR SIZE			
	**< 2cm Ø**	**> 2cm Ø**	
	*n = 18*	*n = 48*	
*SNAI1*	0.169 ± 0.125	0.134 ± 0.043	0.367
*SNAI2*	0.301 ± 0.201	0.351± 0.122	0.835
*ESRP1*	0.662 ± 0.122	0.665 ± 0.202	0.989
E-cadherin	1.431 ± 0.234	1.240 ± 0.137	0.486
*RORC*	1.549 ± 0.322	1.970 ± 0.449	0.450
*VIM*	5.831 ± 2.940	7.003 ± 1.995	0.743
N-cadherin	1.686 ± 0.335	2.576 ± 0.576	0.187
*TWIST*	0.025 ± 0.014	0.053 ± 0.028	0.378
EXTRASELLAR GROWTH			
	**NO**	**YES**	
	*n = 11*	*n = 60*	
*SNAI1*	0.050 ± 0.019	0.152 ± 0.051	**0.049**
*SNAI2*	0.129 ± 0.044	0.356 ± 0.116	0.060
*ESRP1*	0.549 ± 0.118	0.665 ± 0.167	0.579
E-cadherin	2.068 ± 1.265	1.228 ± 0.919	**0.047**
*RORC*	1.889 ± 0.235	1.924 ± 0.377	0.938
*VIM*	2.795 ± 0.805	7.011 ± 1.836	**0.039**
N-cadherin	2.046 ± 0.208	2.414± 0.480	0.484
*TWIST*	0.016 ± 0.011	0.048 ± 0.023	0.209
			
SINUS INVASION			
	**NO**	**YES**	
	*n = 33*	*n = 34*	
*SNAI1*	0.160 ± 0.074	0.124 ± 0.054	0.693
*SNAI2*	0.323 ± 0.144	0.341 ± 0.147	0.933
*ESRP1*	0.482 ± 0.084	0.833 ± 0.279	0.236
E-cadherin	1.326 ± 0.198	1.379 ± 0.156	0.835
*RORC*	1.413 ± 0.206	2.378 ± 0.620	0.147
*VIM*	5.627 ± 2.502	7.188 ± 2.528	0.633
N-cadherin	1.729 ± 0.215	2.950 ± 0.801	0.149
*TWIST*	0.029 ± 0.014	0.059 ± 0.037	0.453
PRESURGICAL HEADACHE			
	**NO**	**YES**	
	*n = 41*	*n = 31*	
*SNAI1*	0.054 ± 0.021	0.238 ± 0.092	**0.050**
*SNAI2*	0.147 ± 0.056	0.536 ± 0.205	0.076
*ESRP1*	0.720 ± 0.239	0.541 ± 0.093	0.488
E-cadherin	1.471 ± 0.165	1.263 ± 0.177	0.391
*RORC*	2.110 ± 0.499	1.663 ± 0.330	0.458
*VIM*	4.109 ± 1.523	9.084 ± 2.886	0.134
N-cadherin	2.529 ± 0.586	2.105 ± 0.525	0.592
*TWIST*	0.016 ± 0.009	0.076 ± 0.042	0.084
PRESURGICAL HYPOPITUITARISM			
	**NO**	**YES**	
	*n = 39*	*n = 33*	
*SNAI1*	0.066 ± 0.024	0.230 ± 0.092	0.095
*SNAI2*	0.154 ± 0.073	0.539 ± 0.200	0.078
*ESRP1*	0.553 ± 0.089	0.794 ± 0.304	0.453
E-cadherin	1.402 ± 0.177	1.299 ± 0.175	0.679
*RORC*	1.631 ± 0.284	2.274 ± 0.642	0.365
*VIM*	3.223 ± 1.092	10.092 ± 3.175	**0.048**
N-cadherin	1.987 ± 0.451	2.820 ± 0.742	0.346
*TWIST*	0.014 ± 0.005	0.080 ± 0.042	0.132
VISUAL ALTERATIONS			
	**NO**	**YES**	
	*n = 43*	*n = 29*	
*SNAI1*	0.069 ± 0.025	0.175 ± 0.067	0.145
*SNAI2*	0.274 ± 0.168	0.344 ± 0.119	0.737
*ESRP1*	0.620 ± 0.152	0.655 ± 0.207	0.895
E-cadherin	1.402 ± 0.177	1.299 ± 0.175	0.679
*RORC*	2.017 ± 0.667	1.852 ± 0.309	0.823
*VIM*	4.552 ± 1.709	7.342 ± 2.254	0.327
N-cadherin	2.081 ± 0.559	2.506 ± 0.550	0.590
*TWIST*	0.019 ± 0.009	0.057 ± 0.030	0.238

P-value was calculated for each tumor characteristic (Student’s t test or Mann–Whitney U test, as appropriate). Significant p-values are shown in bold. SE, Standard Error.

### 3.3 NF-PitNETs showed a more mesenchymal expression profile than GH-secreting tumors

**Figure 1:**
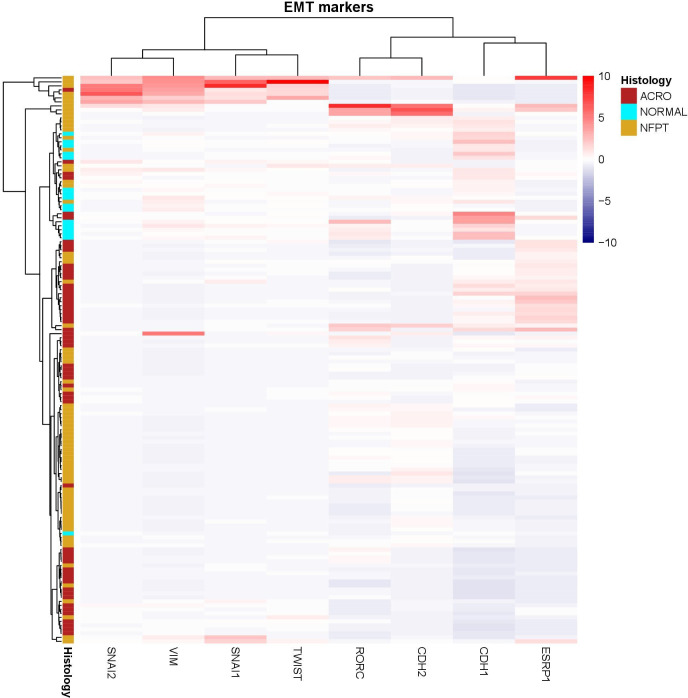
Dendrogram and unsupervised hierarchical clustering heatmap of the EMT-related genes in NF-PitNETs (NFPT), somatotropinomas (ACRO) and healthy pituitary tissue (NORMAL) using Ward’s minimum variance method and Minkowski distance.

We recently reported that most GH-secreting pituitary tumors showed a hybrid and variable EMT expression profile rather than a clear binarial epithelial or mesenchymal phenotype (23). Taking these data into account, we compared the expression of EMT-related markers in GH-secreting tumors, NF-PitNETs and normal pituitaries. Unsupervised hierarchical clustering analysis revealed multiple EMT transition states, with normal pituitaries forming a subcluster associated with high levels of the epithelial marker E-cadherin while NF-PitNETs and GH-secreting tumors were mixed in several different subclusters. Interestingly, a cluster associated with a mesenchymal expression profile was found in 1 GH-secreting adenoma and 6 NF-PitNETs (**Figure 1**). This result showed that 12.5% of NF-PitNETs harbored an expression profile compatible with an advanced EMT transformation compared to 3.6% of GH-secreting tumors.

### 3.4 Association of SRLs and dopaminergic response markers with clinical variables in NF-PitNETs: *PEBP1* as a predictor of recurrence

We also analyzed the expression of SRLs response biomarkers in NF-PitNETs. In those patients in which clinical information was available, gene expression was correlated with clinical parameters (**Table 3**). Tumor size showed a negative association with the *DRD2* long isoform (Pearson’s r = -0.25, p = 0.05). Extrasellar extension was related to *SSTR2, PEBP1*, Ki-67 and *KLK10* (p = 0.031, p=0.033, p =0.042 and p = 0.008, respectively).

**Table 3:** Association between the relative expression of each SRLs response marker and different tumor characteristics.

Gene	Relative expression mean ± SE		P-value
TUMOR SIZE			
	**< 2cm Ø**	**> 2cm Ø**	
	*n = 18*	*n = 48*	
*SSTR2*	0.144 ± 0.049	0.119 ± 0.034	0.660
*SSTR3*	0.621 ± 0.372	0.571± 0.129	0.911
*SSTR5*	0.258 ± 0.229	0.007 ± 0.004	0.288
*DRD2* short isoform	2.706 ± 0.928	1.434 ± 0.171	0.096
*DRD2* long isoform	2.908 ± 0.738	1.692 ± 0.170	**0.046**
*ARRB1*	0.845 ± 0.211	0.743 ± 0.113	0.716
*PLAGL1*	0.578 ± 0.181	0.498 ± 0.193	0.752
*PEBP1*	18.016 ± 1.411	16.920 ± 1.139	0.577
Ki-67	0.034 ± 0.009	0.060 ± 0.016	0.081
*AIP*	1.913 ± 0.017	2.066 ± 0.097	0.398
IN1-GHRL	0.080 ± 0.057	0.024 ± 0.002	0.348
*KLK10*	0.010 ± 0.007	0.017 ± 0.004	0.413
*GHRL*	0.055 ± 0.017	0.032 ± 0.005	0.232
EXTRASELLAR GROWTH			
	**NO**	**YES**	
	*n = 11*	*n = 60*	
*SSTR2*	0.054 ± 0.016	0.132 ± 0.031	**0.031**
*SSTR3*	0.454 ± 0.092	0.611± 0.152	0.379
*SSTR5*	0.010 ± 0.009	0.083 ± 0.070	0.306
*DRD2* short isoform	1.683 ± 0.360	1.850 ± 0.322	0.731
*DRD2* long isoform	2.212 ± 0.459	2.107 ± 0.459	0.848
*ARRB1*	0.605 ± 0.090	0.786 ± 0.111	0.209
*PLAGL1*	0.412 ± 0.235	0.500 ± 0.160	0.760
*PEBP1*	14.673 ± 1.372	17.977 ± 1.026	**0.033**
Ki-67	0.028 ± 0.007	0.054 ± 0.013	**0.042**
*AIP*	2.041 ± 0.145	2.050 ± 0.088	0.959
IN1-GHRL	0.025 ± 0.005	0.041 ± 0.018	0.365
*KLK10*	0.005 ± 0.001	0.017 ± 0.004	**0.008**
*GHRL*	0.029 ± 0.008	0.039 ± 0.007	0.347
SINUS INVASION			
	**NO**	**YES**	
	*n = 33*	*n = 34*	
*SSTR2*	0.115 ± 0.038	0.133 ± 0.039	0.748
*SSTR3*	0.523 ± 0.198	0.672 ± 0.176	0.576
*SSTR5*	0.135 ± 0.122	0.012 ± 0.006	0.317
*DRD2* short isoform	1.967 ± 0.516	1.585 ± 0.214	0.497
*DRD2* long isoform	2.292 ± 0.416	1.796 ± 0.218	0.295
*ARRB1*	0.783 ± 0.155	0.706 ± 0.110	0.686
*PLAGL1*	0.561 ± 0.230	0.425 ± 0.176	0.642
*PEBP1*	17.495 ± 1.484	16.523 ± 0.978	0.587
Ki-67	0.040 ± 0.015	0.059 ± 0.018	0.422
*AIP*	2.141 ± 0.125	1.941 ± 0.094	0.207
IN1-GHRL	0.054 ± 0.030	0.023 ± 0.002	0.319
*KLK10*	0.011 ± 0.004	0.020 ± 0.006	0.194
*GHRL*	0.031 ± 0.008	0.038 ± 0.007	0.475
PRESURGICAL HEADACHE			
	**NO**	**YES**	
	*n = 41*	*n = 31*	
*SSTR2*	0.094 ± 0.024	0.147 ± 0.051	0.350
*SSTR3*	0.468 ± 0.076	0.731 ± 0.273	0.360
*SSTR5*	0.010 ± 0.004	0.148 ± 0.133	0.307
*DRD2* short isoform	1.958 ± 0.231	1.676 ± 0.533	0.641
*DRD2* long isoform	2.385 ± 0.275	1.816 ± 0.424	0.265
*ARRB1*	0.590 ± 0.067	0.975 ± 0.189	0.063
*PLAGL1*	0.367 ± 0.257	0.644 ± 0.257	0.349
*PEBP1*	17.877 ± 1.098	16.731 ± 1.455	0.532
Ki-67	0.036 ± 0.008	0.068 ± 0.023	0.203
*AIP*	2.047 ± 0.093	2.042 ± 0.127	0.974
IN1-GHRL	0.023 ± 0.002	0.059 ± 0.033	0.289
*KLK10*	0.012 ± 0.004	0.017 ± 0.005	0.480
*GHRL*	0.036 ± 0.007	0.059 ± 0.009	0.784
PRESURGICAL HYPOPITUITARISM			
	**NO**	**YES**	
	*n = 39*	*n = 33*	
*SSTR2*	0.155 ± 0.048	0.087 ± 0.019	0.201
*SSTR3*	0.695 ± 0.090	0.454 ± 0.090	0.347
*SSTR5*	0.133 ± 0.004	0.006 ± 0.004	0.276
*DRD2* short isoform	2.162 ± 0.489	1.547 ± 0.247	0.267
*DRD2* long isoform	2.338 ± 0.414	1.983 ± 0.271	0.476
*ARRB1*	0.606 ± 0.086	0.571 ± 0.175	0.181
*PLAGL1*	0.482 ± 0.114	0.511 ± 0.280	0.924
*PEBP1*	18.383 ± 1.269	16.528 ± 1.360	0.322
Ki-67	0.042 ± 0.016	0.057 ± 0.018	0.526
*AIP*	2.084 ± 0.119	2.001 ± 0.104	0.599
IN1-GHRL	0.051 ± 0.029	0.026 ± 0.003	0.390
*KLK10*	0.011 ± 0.004	0.019 ± 0.006	0.256
*GHRL*	0.035 ± 0.008	0.036 ± 0.007	0.961
VISUAL ALTERATIONS			
	**NO**	**YES**	
	*n = 43*	*n = 29*	
*SSTR2*	0.134 ± 0.048	0.107 ± 0.030	0.635
*SSTR3*	0.343 ± 0.075	0.724 ± 0.193	0.071
*SSTR5*	0.013 ± 0.006	0.104 ± 0.092	0.328
*DRD2* short isoform	1.642 ± 0.294	1.953 ± 0.396	0.531
*DRD2* long isoform	2.061 ± 0.382	2.187 ± 0.313	0.799
*ARRB1*	0.662 ± 0.146	0.812 ± 0.119	0.428
*PLAGL1*	0.671 ± 0.289	0.375 ± 0.132	0.359
*PEBP1*	17.078 ± 1.162	17.566 ± 1.234	0.774
Ki-67	0.045 ± 0.019	0.052 ± 0.014	0.774
*AIP*	1.932 ± 0.110	2.113 ± 0.101	0.228
IN1-GHRL	0.021 ± 0.002	0.048 ± 0.023	0.249
*KLK10*	0.011 ± 0.006	0.017 ± 0.004	0.409
*GHRL*	0.039 ± 0.008	0.036 ± 0.007	0.828

P-value was calculated for each tumor characteristic (Student's t test or Mann–Whitney U test, as appropriate). Significant p-values are shown in bold. SE, Standard Error.

**Figure 2:**
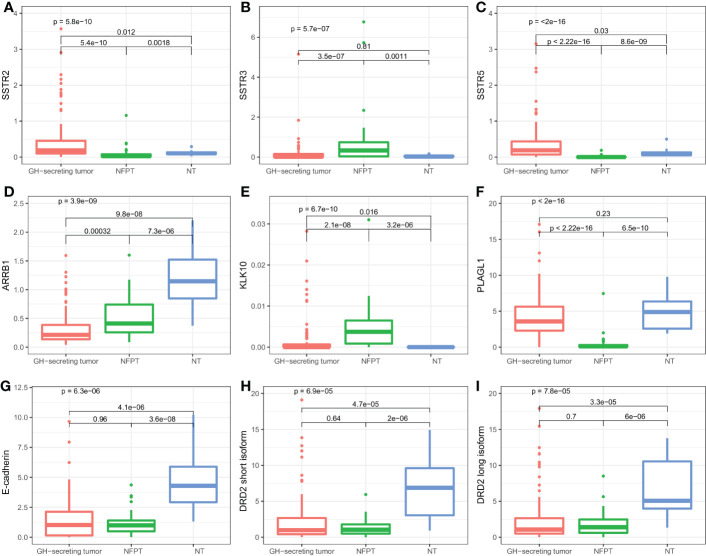
Boxplots of SRL-related genes according to the different histological samples. Relative expression of *SSTR2* **(A)**, *SSTR3* **(B)**, *SSTR5* **(C)**, *ARRB1* **(D)**, *KLK10* **(E)**, *PLAGL1* **(F)**, E-cadherin **(G)**, *DRD2* short **(H)** and long **(I)** isoform. NT, Normal tissue.

Differences were found also in SRLs biomarkers between NF-PitNETs, GH-secreting pituitary adenomas and non-tumoral pituitaries. GH-secreting tumors showed higher levels of *SSTR2* and *SSTR5*, whereas *SSTR3* was more expressed in NF-PitNETs (**Figures 2A–C**). *ARRB1* levels were higher in normal tissue (**Figure 2D**). On the other hand, NF-PitNETs seem to be characterized by high levels of *KLK10* and *PLAGL1* (**Figures 2E, F**). Finally, non-tumoral tissue presented high levels of E-cadherin and *DRD2* (**Figures 2G–I**).

**Figure 3:**
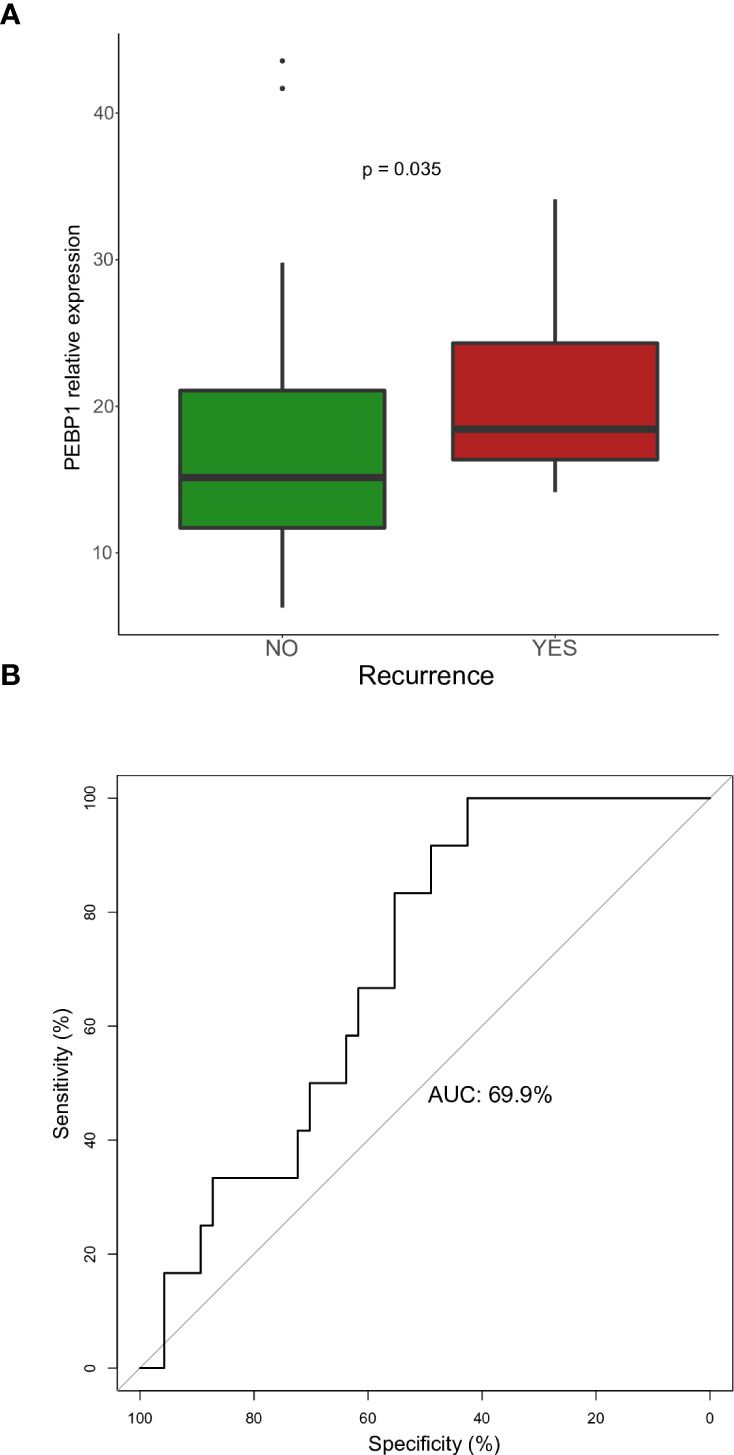
**(A)** Relative expression of *PEBP1* in NF-PitNETs in tumors that recurred vs. tumors that did not. **(B)** ROC curve for predicting recurrence using *ARRB1* expression in NF-PitNETs.

Furthermore, we wanted to investigate if some of these genes could be used to predict patient’s outcome. We investigated the ability of these markers in predicting recurrence in NF-PitNETs (**Table 4**), and found that only *PEBP1* showed a difference between tumors that recurred and tumors that did not (p=0.036) (**Figure 3A**). When performing a binomial logistic regression, *PEBP1* showed an AUC of 69.9%, for a cut-off of 14.0, with a sensitivity of 100% and a specificity of 42.6% (**Figure 3B**).

**Table 4:** Association between the relative expression of each gene and the development of a recurrence.

Gene	Relative expression mean ± SE		P-value
RECURRENCE			
	**NO**	**YES**	
	*n = 47*	*n = 12*	
*SNAI1*	0.128± 0.053	0.183 ± 0.137	0.713
*SNAI2*	0.364 ± 0.140	0.257± 0.141	0.594
*ESRP1*	0.553 ± 0.103	0.495 ± 0.100	0.688
E-cadherin	1.433 ± 0.161	1.571 ± 0.278	0.673
*RORC*	2.020 ± 0.466	1.600 ± 0.307	0.455
*VIM*	5.888 ± 1.719	8.037 ± 4.541	0.665
N-cadherin	2.586 ± 0.593	1.632 ± 0.307	0.158
*TWIST*	0.025 ± 0.011	0.120 ± 0.101	0.368
*SSTR2*	0.131 ± 0.036	0.095 ± 0.053	0.581
*SSTR3*	0.645 ± 0.186	0.454 ± 0.141	0.418
*SSTR5*	0.098 ± 0.088	0.018 ± 0.016	0.374
*DRD2* short isoform	1.834 ± 0.377	1.878 ± 0.545	0.948
*DRD2* long isoform	2.075 ± 0.303	2.357 ± 0.714	0.721
*ARRB1*	0.775 ± 0.128	0.921 ± 0.206	0.554
*PLAGL1*	0.463 ± 0.173	0.328 ± 0.125	0.531
*PEBP1*	16.809 ± 1.153	20.827 ± 1.805	**0.036**
Ki-67	0.056 ± 0.016	0.038 ± 0.010	0.348
*AIP*	2.075 ± 0.104	2.184 ± 0.078	0.407
IN1-GHRL	0.045 ± 0.022	0.030 ± 0.006	0.509
*KLK10*	0.016 ± 0.005	0.014 ± 0.008	0.762
*GHRL*	0.034 ± 0.007	0.058 ± 0.017	0.215

P-value was calculated for each tumor characteristics (Student’s t test or Mann–Whitney U test, as appropriate). Significant p-values are shown in bold. SE, Standard Error.

### 3.5 Relative expression of drug receptors: *DRD2* is the most expressed receptor both in GH-secreting tumors and NF-PitNETs

**Figure 4:**
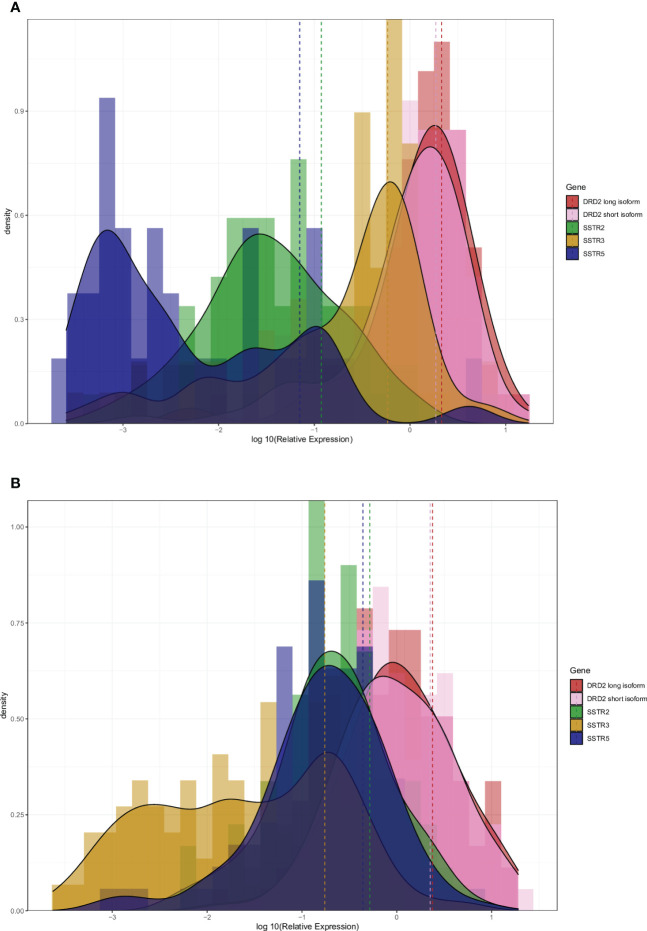
Histogram and density plot of the log_10_ relative expression of *DRD2* long and short isoform, *SSTR2*, *SSTR3* and *SSTR5* in NF-PitNETs **(A)** and GH-secreting pituitary neuroendocrine tumors **(B)**. Dashed line showed the mean expression of each gene.

We wanted to compare relative quantities of drug receptor expression in both NF-PitNETs and GH-secreting tumors. In NF-PitNETs the more prevalent receptor was DRD2, followed by SSTR3, while SSTR2 and SSTR5 presented a low expression (**Figure 4A**). In the case of GH-secreting tumors DRD2 was also the most quantitatively expressed, SSTR2 and SSTR5 were expressed at the same level as the SSTR3 in NF-PitNETs, while SSTR3 showed a very low expression level (**Figure 4B**).

### 3.6 Specific expression profile associated with NF-PitNET cell subtype: low levels of *SSTR3* and *ARRB1* in silent corticotroph adenomas

**Figure 5:**
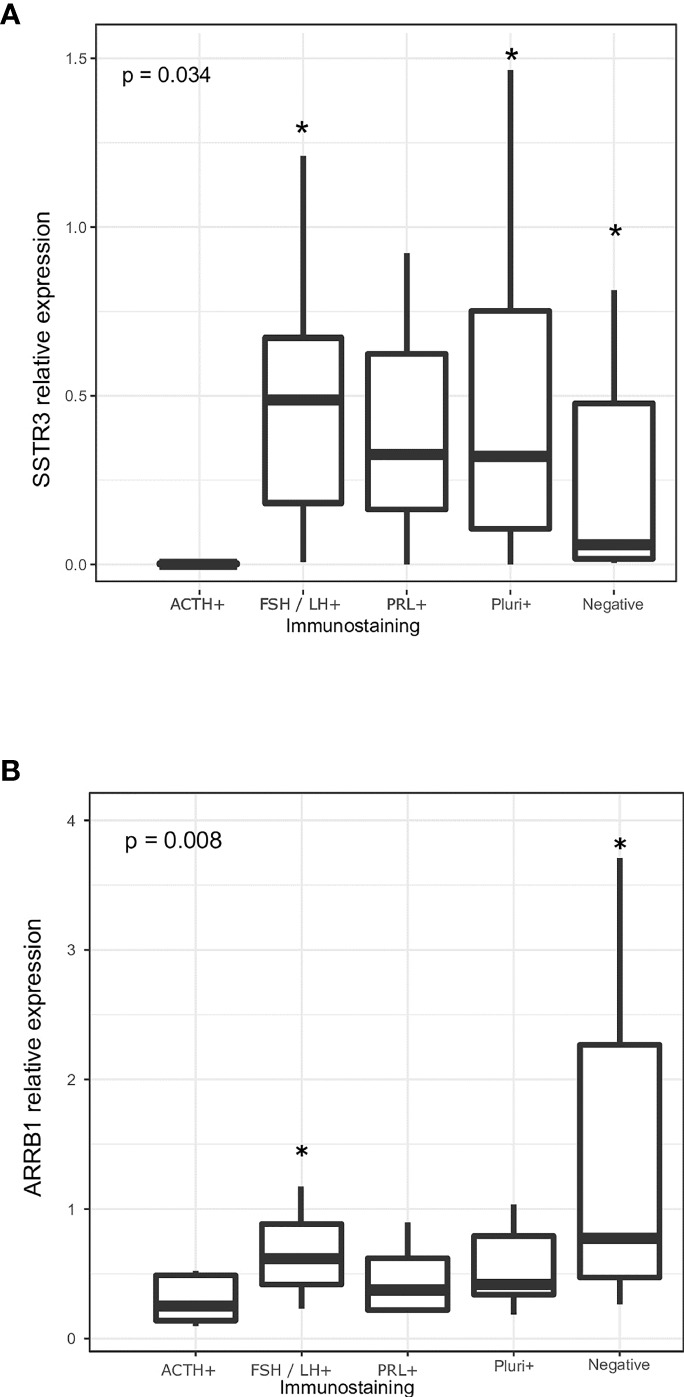
**(A)** Relative expression of *SSTR3* according to the immunohistochemical expression of the pituitary hormones. **(B)** Relative expression of *ARRB1* according to the immunohistochemical expression of the pituitary hormones. P-values for the different group comparisons regarding *SSTR3* expression: ACTH+ vs. FSH/LH-+ (p=0.001), ACTH+ vs. PRL+ (p=0.418), ACTH+ vs. plurihormonal NF-PitNETs (p=0.005), ACTH+ vs. negative tumors (p=0.004), FSH/LH+ vs. PRL+ (p=0.418), FSH/LH+ vs. plurihormonal NF-PitNETs (p=0.429), FSH/LH+ vs. negative tumors (p=0.603), PRL+ vs. plurihormonal NF-PitNETs (p=1), PRL+ vs. negative tumors (p=0.795) and plurihormonal NF-PitNETs vs. negative tumors (p=0.787). P-values for the different group comparisons regarding *ARRB1* expression: ACTH+ vs. FSH/LH-+ (p=0.005), ACTH+ vs. PRL+ (p=0.509), ACTH+ vs. plurihormonal NF-PitNETs (p=0.152), ACTH+ vs. negative tumors (p=0.012), FSH/LH+ vs. PRL+ (p=0.119), FSH/LH+ vs. plurihormonal NF-PitNETs (p=0.085), FSH/LH+ vs. negative tumors (p=0.147), PRL+ vs. plurihormonal NF-PitNETs (p=0.535), PRL+ vs. negative tumors (p=0.051) and plurihormonal NF-PitNETs vs. negative tumors (p=0.026). * indicates the subtypes of NF-PitNETs that showed significant differences compared to ACTH+ NF-PitNETs.

We correlated levels of EMT and SRL response gene expression with immunohistochemical characteristics of NF-PitNETs (**Table 5**). We found differences in *SSTR3* comparing ACTH-expressing NF-PitNETs versus FSH/LH-positive, plurihormonal NF-PitNETs and negative tumors (p = 0.001, p = 0.005 and p = 0.004, respectively) (**Figure 5A**). We also found differences in *ARRB1* comparing ACTH-expressing NF-PitNETs versus FSH/LH-positive and negative cell tumors (p=0.005 and p=0.012, respectively) (**Figure 5B**).

**Table 5:** Analysis of differences between the relative expression of each gene and the positive immunostaining for pituitary hormones.

Gene	P-value
*SNAI1*	0.379
*SNAI2*	0.098
*ESRP1*	0.621
E-cadherin	0.135
*RORC*	0.232
*VIM*	0.333
N-cadherin	0.187
*TWIST*	0.284
*SSTR2*	0.356
*SSTR3*	**0.034**
*SSTR5*	0.754
*DRD2* short isoform	0.359
*DRD2* long isoform	0.670
*ARRB1*	**0.018**
*PLAGL1*	0.060
*PEBP1*	0.455
Ki-67	0.074
*AIP*	0.059
IN1-GHRL	0.676
*KLK10*	0.230
*GHRL*	0.633

P-value was calculated using Kruskal–Wallis test or ANOVA test, as appropriate. Significant P-values are shown in bold.

## 4 Discussion

NF-PitNET is a very unspecific tumor class in which by definition, no clinically detectable hypersecretory pituitary tumors are included. Its main clinical feature is the mass effect upon the surrounding anatomical sellar structures. In the present work we aimed to explore the molecular knowledge of this tumor class and confirmed its very heterogeneous nature. Regarding tumors with extrasellar growth, we found that both EMT and SRL response genes presented differential expression compared to non-invasive tumors. It has already been described that EMT genes correlate with invasive pituitary tumors as well as with angiogenesis and extracellular matrix degrading genes (34). However, we also found that *SSTR2* and *PEBP1* were associated to extrasellar growth in some NF-PitNETs that, as far as we know, has not been previously reported.

The measurement of DA receptor and SRL response genes in NF-PitNETs provides interesting information that could be useful in the case that a pharmacological treatment is considered in this type of pituitary tumors (35); thus, it would be recommendable to perform such studies rather than initiating a blind course of treatment with unpredictable results. In the present study, we found a striking NF-PitNET subtype clustering by using the expression of a few genes. Our results indicate the existence of substantial differences in the SRLs biomarkers’ landscape when NF-PitNETs are compared with somatotropinomas, the latter being an archetype of medically treated pituitary tumors. We believe that this is a relevant finding, since usually the detection of differences in clustering between different types of pituitary tumors requires genome-wide data (36).

Furthermore, the present work can shed light on the controversy over the quantitative analysis of dopamine receptors, somatostatin receptor subtypes and downstream effectors in NF-PitNETs (14, 35, 37, 38).

Another interesting finding of the present study, probably the one of most clinical relevance for its application in personalized medicine, is the association of EMT-markers with local growth in NF-PitNETs. Although this association has been previously reported (18, 39–41), we could observe that the EMT phenomenon is more present in the NF-PitNETs compared to somatotropinomas. Furthermore, EMT has been described as a cause of resistance to medical treatment in pituitary tumors, in particular to SRLs (20); thus, the evaluation of these biomarkers in NF-PitNETs would seem highly recommendable in case of considering medical treatment.

The absence of *SSTR2*, *SSTR5* and *PLAGL1* has also been proposed by other groups as an explanation for the lack of response to SRLs in NF-PitNETs (35, 37). Furthermore, regarding *SSTRs, SSTR3* ligands could be a very interesting druggable target in NF-PitNETs and its pharmacologic activation may be of potential benefits regarding the prevention of tumor regrowth, since *SSTR3* inhibits cell cycle dynamics and promotes apoptosis. A recent experimental study has demonstrated that *SSTR3* monoligands activate signaling mechanisms that reduce NF-PitNET cell viability and inhibit pituitary tumor growth in animal models expressing *SSTR3*, suggesting that it could be an efficacious treatment for NF-PitNETs (42).

When comparing the relative expression of the drug receptors, we found that *DRD2* was the most expressed receptor in both NF-PitNETs and GH-secreting tumors. However, dopaminergic agents are only useful in 30% of tumors in attaining a clinically significant volume reduction (13), while in GH-secreting pituitary tumors, SRLs induce tumor shrinkage in more than 50% of the cases (43, 44). If we assume that the effects of the drugs are correlated with the expression of their target receptors, we would expect beneficial results from targeting *SSTR3* in NF-PitNETs at least at similar levels as observed when targeting *SSTR2* in acromegaly. However, if according to our results *SSTR3* could be an interesting target for most NF-PitNETs, silent corticotroph tumors should be excluded for such a treatment, because of the low receptor levels they express, and therefore no positive results of SRLs treatment would be expectable in this tumor subtype. This is an important issue in order to identify those tumors presenting a worse prognosis, as is the case for this corticotrophic subtype (45). However, the assumption that the effects of drugs are correlated with the levels of expression of their target receptors is too naïve. The presence of target receptors for both DA and SRLs that act *via* the MAPK pathway are a sine quanon requirement for a positive therapy outcome. However, the precise molecular mechanisms and the modulatory effect of different transcription factors, intermediate effectors and even cytoskeleton proteins are still not clear (46). The assumption that the presence of more target receptors means more drug efficacy is not so straightforward (47).

Moreover, we found an association between *KLK10* expression in NF-PitNETs and extrasellar extension. *KLK10* is a gene encoding for a serine protease, member of the tissue kallikrein proteins (KLKs) (48). KLKs are widely recognized as cancer biomarkers (49) and are considered interesting targets for the development of novel drugs (50). *KLK10* has been involved in the development of many cancers, such as ovarian (51), breast (52), prostate (53) and thyroid (54) cancers, although the specific role of *KLK10* in tumorigenesis is not yet sufficiently defined. Regarding human pituitary tumors, *KLK10* has been found to be consistently expressed in prolactinomas, thyrotropinomas, somatotrophinomas and corticotroph adenomas (55, 56). *KLK10* could thus be an interesting target for NF-PitNETs, but currently more studies on its pathophysiologic and mechanistic implications should be performed, either silencing or overexpressing its gene in order to clearly define its implications in tumor growth as well as its potential therapeutic use.

Finally, we also found that recurrent tumors expressed higher levels of *PEBP1*. *PEBP1*, also known as *RKIP*, is considered a metastasis suppressor gene (45, 57). In addition, it has been linked to poor response to SRLs in acromegaly (58). We found that *PEBP1* is a potential biomarker for predicting recurrence, due to its high sensitivity, although its low specificity may limit its usefulness in clinical practice. Thus, with the current lack of robust biomarkers for implementation of predictive medicine in NF-PitNETs, *PEBP1* may be a first step to classify a subgroup of these tumors requiring a more careful follow-up. A validation study of this and the other biomarkers presented in the present work is required. Moreover, drugs specifically designed to target *KLK10* and *PEBP1* would be welcome, and would be useful for pituitary tumors currently lacking an efficient medical treatment.

In summary, the lack of response to SRLs and DA in NF-PitNETs could be partly explained by a more common EMT phenomenon in this subset of pituitary tumors than in functioning ones (20). Moreover, despite the fact that *SSTR3* could be a good therapeutic target, it will presumably not be effective in silent corticotroph tumors. The absence of validated prognostic markers or a prognostic classification for NF-PitNETs limits the evaluation of medical strategies for these lesions. Different pathological markers have been suggested so far, including those of the present study. Their prognostic value should be prospectively confirmed in a large multicenter study which would help to build the instrument to implement precision medicine also for NF-PitNETs.

## Data availability statement

The raw data supporting the conclusions of this article will be made available by the authors, without undue reservation.

## Ethics statement

The studies involving human participants were reviewed and approved by Germans Trias i Pujol Hospital Ethical Committee for Clinical Research (Ref.: EO-11-080 http://www.ceicgermanstrias.cat/index.html). The patients/participants provided their written informed consent to participate in this study.

## Author contributions

JG, MM-P, MJ and MP-D contributed to conception and design of the study. MM-P organized the database. JG performed the statistical analysis, performed the experiments and wrote the first draft of the manuscript. BB, MJ and MP-D wrote sections of the manuscript. The remaining authors provided patients and clinical information to the study and critically discussed the results. All authors contributed to the article and approved the submitted version.
